# Berberine-induced apoptotic and autophagic death of HepG2 cells requires AMPK activation

**DOI:** 10.1186/1475-2867-14-49

**Published:** 2014-06-11

**Authors:** Rong Yu, Zhi-qing Zhang, Bin Wang, Hong-xin Jiang, Lei Cheng, Li-ming Shen

**Affiliations:** 1 Department of Oncology, Suzhou Municipal Hospital, the Affiliated Suzhou Hospital of Nanjing Medical University, Suzhou, Jiangsu 215000, China; 2 Department of Interventional Radiology, Suzhou Municipal Hospital, the Affiliated Suzhou Hospital of Nanjing Medical University, Suzhou, Jiangsu 215000, China; 3 Institute of Neuroscience, Soochow University, Suzhou, Jiangsu 215123, China

**Keywords:** Hepatocellular carcinoma, Berberine, AMPK, Apoptosis, Autophagy and mTOR

## Abstract

**Background:**

Hepatocellular carcinoma (HCC), the primary liver cancer, is one of the most malignant human tumors with extremely poor prognosis. The aim of this study was to investigate the anti-cancer effect of berberine in a human hepatocellular carcinoma cell line (HepG2), and to study the underlying mechanisms by focusing on the AMP-activated protein kinase (AMPK) signaling cascade.

**Results:**

We found that berberine induced both apoptotic and autophagic death of HepG2 cells, which was associated with a significant activation of AMPK and an increased expression of the inactive form of acetyl-CoA carboxylase (ACC). Inhibition of AMPK by RNA interference (RNAi) or by its inhibitor compound C suppressed berberine-induced caspase-3 cleavage, apoptosis and autophagy in HepG2 cells, while AICAR, the AMPK activator, possessed strong cytotoxic effects. In HepG2 cells, mammalian target of rapamycin complex 1 (mTORC1) activation was important for cell survival, and berberine inhibited mTORC1 via AMPK activation.

**Conclusions:**

Together, these results suggested that berberine-induced both apoptotic and autophagic death requires AMPK activation in HepG2 cells.

## Background

Hepatocellular carcinoma (HCC), the primary liver cancer, is one of the most malignant human tumors with extremely poor prognosis [1]. HCC accounts for over 80% of all liver cancers and is diagnosed in over 600,000 people annually [1]. HCC has become one of the leading causes of cancer-related mortality in the United States and around the world [1,2]. There is currently no clinically proved curable therapy for the advanced HCC [1,3], and a large percentage of advanced HCC do not respond to any chemotherapies, mainly due to the high level of intrinsic and acquired chemo-resistances [4]. Thus, the development of novel and effective therapeutic approaches for this devastating disease is of utmost relevance [3,4].

Berberine, the isoquinoline alkaloid presented in Huanglian (Coptis chinensis) and many Chinese medicinal herbs, has shown significant anti-tumor activities both *in vitro* and *in vivo*[5]. Its high anti-cancer efficiency is associated with its transcriptional and post-transcriptional regulation of some oncogenes and carcinogenesis-related genes, and its interactions with both DNA and RNA [5]. In the current study, we aimed to investigate the anti-cancer ability of berberine in a human HCC line (HepG2), and to study the underlying mechanisms by focusing on the AMP-activated protein kinase (AMPK) signaling cascade.

Under the metabolic stress conditions such as hypoxia, heat shock, oxidative stress, and exercise where ATP is depleted, AMPK is activated and functions as a major metabolic switch to maintain energy homeostasis [6,7]. This highly conserved heterotrimeric kinase has also been shown to act as an intrinsic regulator of mammalian cell cycle [6,7]. Moreover, AMPK plays a important role in cancer cell survival and apoptosis. As a matter of fact, a number of anti-cancer medicinal herb extracts activate AMPK-dependent cell death pathways [8,9]. Recent studies have shown that berberine could also activate AMPK [10,11], however, the potential roles and underlying mechanisms of AMPK in mediating berberine-induced cancer cell death remain largely unknown. In this study, we found that AMPK activation is important for berberine-induced both apoptotic and autophagic cell death in HCC HepG2 cells.

## Results

### Berberine inhibits survival and proliferation of HepG2 cells

**Figure 1:**
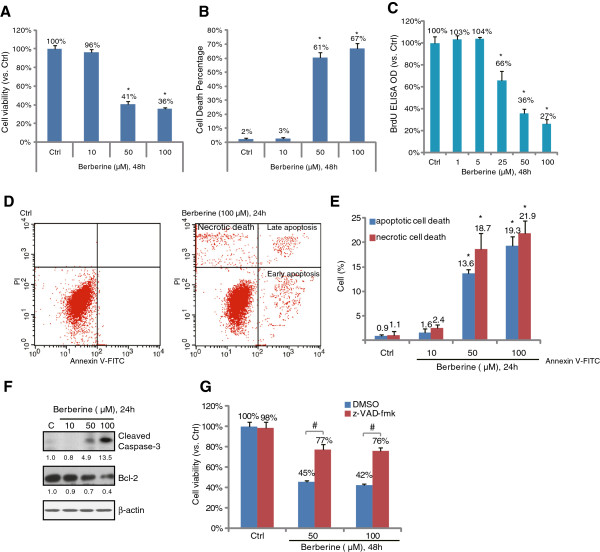
**Berberine induces apoptotic and necrotic death of HepG2 cells*.*** HepG2 cells were either left untreated or treated with described concentration of berberine, cells were further cultured in DMEM for 48 hours, the cell viability was tested by “MTT” assay **(A)**, the percentage of trypan blue dye positive cells was recorded **(B)**; HepG2 cell proliferation was analyzed by BrdU incorporation assay **(C)**. HepG2 cells treated with or without berberine were cultured in DMEM for 24 hours, apoptotic and necrotic cell death was tested by Annexin V FACS assay **(D and E)**, expressions of cleaved-caspase 3, Bcl-2 and β-actin were tested by western blots **(F)**. HepG2 cells were pre-treated with z-VAD-fmk (50 μM) for 1 hour, followed by berberine (50 and 100 μM) stimulation, cells were further cultured for 48 hours before cell viability was tested **(G)**. Experiments in this figure were repeated three times, and similar results were obtained. Data were expressed as mean ± SD. *p < 0.05 vs. Ctrl group **(A and B)**. ^#^***p*** < 0.05 vs. berberine-treated group **(C)**.

First we examined the effect of berberine on HepG2 cell survival and proliferation. Cell viability “MTT” assay was performed. Results in Figure 1A clearly showed that high-dose of berberine (50 and 100 μM) dramatically inhibited HepG2 cell survival, as the MTT OD decreased significantly. Meanwhile, the number of trypan blue positive (“dead”) cells increased sharply after high dose of berberine stimulation (50 and 100 μM) (Figure 1B). Interestingly, a relative low dose of berberine (10 μM) had almost no effects on HepG2 cell survival (Figure 1A and B). We also examined the effect of berberine on HepG2 cell proliferation. Using the BrdU incorporation assay, we demonstrated that berberine dose-dependently suppressed HepG2 cell proliferation (Figure 1C). Taken together, these results suggested that berberine significantly inhibits survival and proliferation of HepG2 cells.

### Berberine induces apoptotic and necrotic death of HepG2 cells

The results above showed that berberine inhibited HepG2 cell survival and proliferation; next we tested whether cell apoptosis was involved in such an effect. As shown in Figure 1D and E, berberine (50 and 100 μM) induced both early (Annexin V^+^/PI^−^) and late (Annexin V^+^/PI^+^) apoptosis in HepG2 cells. Meanwhile, berberine also caused caspase-3 cleavage and Bcl-2 degradation (Figure 1F). Interestingly, we noticed that berberine also induced necrotic HepG2 cell death (Annexin V^−^/PI^+^) (Figure 1D and E). Further, cell viability assay results in Figure 1G showed that z-VAD-fmk, the general caspase inhibitor, only suppressed (but not reversed) berberine-induced HepG2 viability loss, indicating that both apoptotic and necrotic death also accounted for berberine-induced cytotoxicity in HepG2 cells.

### Berberine induces autophagic death in HepG2 cells

**Figure 2:**
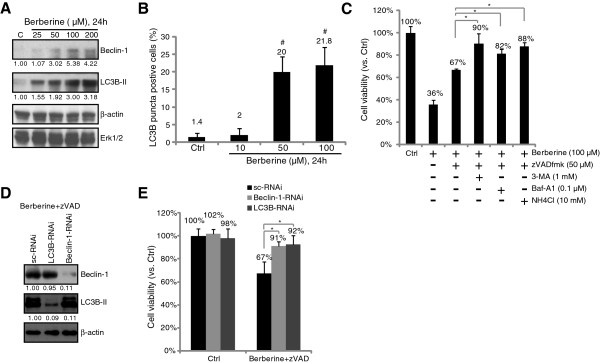
**Berberine induces apoptotic and necrotic death of HepG2 cells*.*** HepG2 cells were either left untreated or treated with described concentration of berberine (10, 50, 100 and 200 μM), cells were further cultured in DMEM (no serum) for 24 hours, expressions of Beclin-1, LC3B-II, Erk1/2 and β-actin were tested by western blots **(A)**. The number of LC3-GFP puncta positive cells (autophagic cells) was counted **(B)**.Cell viability of HepG2 cells with indicated treatment for 48 hours was tested by MTT assay **(C)**. HepG2 cells transfected with scramble control siRNA, Beclin-1 siRNA or LC3B siRNA (100 nM each, for 48 hours) were either left untreated, or stimulated with berberine (100 μM) plus z-VAD-fmk (50 μM) (Berberine + ZVAD), cells were further cultured for 48 hours, expression of Beclin-1, LC3B and β-actin was tested by western blots **(D)**, cell viability was also tested **(E)**. Experiments in this figure were repeated three times, and similar results were obtained. Data were expressed as mean ± SD. ^#^***p*** < 0.05 vs. Ctrl group **(B)**. *p < 0.05 **(C and E)**.

The above results showed that berberine induced both apoptotic and necrotic death of HepG2 cells. Thus, we tested autophagy induction in berberine-treated HepG2 cells. Expressions of Beclin-1 [12,13] and light chain 3 (LC3) B-II, two autophagy indicators, in berberine-treated HepG2 cells were examined. Results in Figure 2A clearly showed that berberine induced Beclin-1 and LC3B-II up-regulation in HepG2 cells. Meanwhile, the number of HepG2 cells with intense LC3B-GFP puncta was increased dramatically after berberine treatment (Figure 2B). In order to explore the role of autophagy in berberine-induced HepG2 cell cytotoxicity, we first utilized caspase inhibitor (z-VAD-fmk) to block cell apoptosis. In this condition, we found that the autophagy inhibitors including 3-methyladenine (3-MA, an inhibitor of class III PI3-kinase), Bafilomycin A1, (Baf A1, a proteolysis inhibitor) and NH_4_Cl (another proteolysis inhibitor) significantly inhibit berberine-induced viability loss (Figure 2C). Further, siRNA-mediated silencing of LC3B or Beclin-1 (Figure 2D) also suppressed berberine-induced HepG2 cell death (Figure 2E). These results suggest that autophagy activation is important for berberine-mediated cytotoxicity.

### Activation of AMPK is involved in berberine-induced cytotoxicity in HepG2 cells

**Figure 3:**
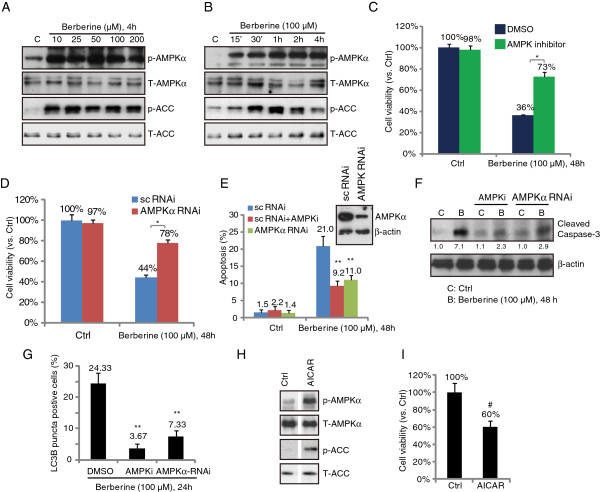
**Activation of AMPK is involved in berberine-induced cytotoxicity in HepG2 cells*.*** HepG2 cells were either left untreated or treated with described concentration of berberine (10, 25, 50, 100 and 200 μM) for 4 hours, or treated with 100 μM of berberine for described time (15′, 30′, 1 h, 2 h and 4 h), phospho- and total AMPKα/ACC were tested by western blots **(A and B)**. HepG2 cells were pre-treated with the AMPK inhibitor compound C (10 μM) for 1 hour, followed by berberine (100 μM) stimulation, cells were further cultured for 48 hours before cell viability was tested **(C)**. Scramble control RNAi or AMPKα RNAi transfected HepG2 cells were either left untreated or treated with berberine (100 μM), cells were further cultured for 48 hours before cell viability was tested **(D)**, expressions of AMPKα and β-actin in those cells were also tested by western blot (D, upper). Above cells were also tested for cell apoptosis 24 hours after stimulation **(E)**, expressions of cleaved-caspase-3 and β-actin were examined **(F)**, the number of LC3-GFP puncta positive cells were also recorded **(G)**. HepG2 cells were either left untreated or treated with AICAR (1 mM), phospho- and total AMPKα/ACC were tested by western blots 2 hours after stimulation (H), and cell viability was examined by MTT assay after 48 hours incubation **(I)**. Experiments in this figure were repeated three times, and similar results were obtained. *p < 0.05 (C and D). **p < 0.05 vs. berberine-treated group **(G and E)**. ^#^***p*** < 0.05 vs. Ctrl group (I).

As shown in Figure 3A and B, berberine-induced significant AMPK activation in HepG2 cells, as the expressions of phosphorylated AMPKα and its downstream ACC in HepG2 cells were significantly increased after berberine treatment (Figure 3A and 3B). Importantly, AMPK inhibition by its inhibitor compound C (AMPKi) or RNA interference (AMPKα-RNAi) suppressed berberine-induced cell viability loss (Figure 3C and D). Meanwhile, berberine-induced apoptosis and caspase-3 cleavage were also inhibited by AMPK inhibition (Figure 3E and F). Further, the AMPK inhibitor or RNAi also reduced the number of LC3-GFP puncta (autophagic) cells after berberine treatment, indicating that AMPK is required for both apoptosis and autophagy induction by berberine. The fact that the AMPK activator 5-aminoimidazole-4-carboxyamide-1-β-D-ribofuranoside (AICAR) (Figure 3H) inhibited HepG2 cell survival (Figure 3I) further confirmed that activation of AMPK is involved in berberine-induced cytotoxicity in HepG2 cells.

### mTORC1 activation is required for HepG2 cell survival, inhibited by berberine

**Figure 4:**
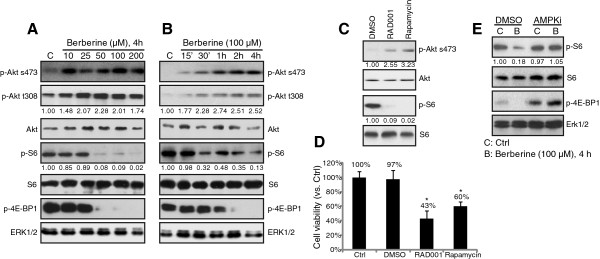
**mTORC1 activation is required for HepG2 cell survival, inhibited by berberine*.*** HepG2 cells were either left untreated or treated with described concentration of berberine (10, 25, 50, 100 and 200 μM) for 4 hours, or treated with 100 μM of berberine for described time, expressions of phospho- and total Akt, S6 and 4EBP1, as well as Erk1/2 were tested by western blots **(A and B)**. HepG2 cells were either left untreated or stimulated with RAD001 (200 nM) or rapamycin (200 nM), Akt and S6 activations were tested by western blots 4 hours after stimulation **(C)**, cell viability was analyzed by MTT assay 48 hours after stimulation **(D)**. HepG2 cells were pre-treated with the AMPK inhibitor compound C (AMPKi, 10 μM) for 1 hour, followed by berberine (100 μM) stimulation for 4 hours, expressions of S6 (p- and t-), p-4E-BP1 and Erk1/2 were tested by western blots **(E)**. Experiments in this figure were repeated three times, and similar results were obtained. Data were expressed as mean ± SD. *p < 0.05 vs. Ctrl group **(D)**.

Activation of Akt and mammalian target of rapamycin complex 1 (mTORC1) signaling plays a key role in liver cancer cell survival, proliferation and apoptosis-resistance; we then examined these signalings in berberine-treated HepG2 cells. Western blot results in Figure 4A and B showed that berberine induced Akt activation in a time and dose-dependently manner in HepG2 cells. Note that Akt activation was reflected by the increased expressions of phospho (p)-Akt (Ser 473 and Thr 308). However, at the same time, berberine significantly inhibited mTORC1 activation in HepG2 cells (Figure 4A and B), as p-S6 and p-4E-BP1 downregulated sharply after high dose of berberine (>50 μM) treatment. mTORC1 inhibition started with 0.5-1 hour after berberine (100 μM) treatment (Figure 4B). These results together suggested that berberine activates Akt while inhibiting mTORC1 in HepG2 cells. Similarly, RAD001 and rapamycin, two mTORC1 inhibitors blocked S6 phosphorylation and activated Akt in HepG2 cells (Figure 4C), these two also inhibited HepG2 cell survival (Figure 4D). Interestingly, berberine-induced mTORC1 inhibition was almost reversed by AMPK inhibitor compound C (Figure 4E), suggesting that AMPK activation was required mTORC1 inhibition by berberine. Together, these results suggested that berberine, via activating AMPK signaling, inhibits mTORC1 activation and cell survival in HepG2 cells. Also, berberine-induced Akt activation is probably through mTOR-dependent feedback pathways [14].

## Discussions

Although AMPK is generally recognized as the metabolic switcher [6], a number of recent papers have suggested that cellular stresses-activated AMPK also promotes cell apoptosis [15], such an effect by AMPK is through regulating AMPK’s downstream signals, including c-Jun N-terminal kinases (JNK) [16], p53 [17] and mTOR [15]. Meanwhile, anti-cancer chemotherapies such as taxol [18,19] and temozolomide [20] activate AMPK-dependent apoptosis pathways. Meanwhile, resveratrol [21], capsaicin [8] and EGCG [22] anti-cancer plant extracts induced cancer cell death also requires AMPK activation. In the current study, we also observed a significant AMPK activation in berberine-treated HepG2 cells. Inhibition of AMPK by RNAi or compound C suppressed berberine-induced caspase-3 cleavage, apoptosis and autophagy in HepG2 cells. Conversely, HepG2 cell viability was inhibited by the AMPK activator AICAR. These results together suggested that AMPK is required for berberine-induced anti-cancer effects in HepG2 cells.

AMPK regulated cell death was, however, not solely rely on apoptosis induction. As a matter of fact, recent studies have indentified another way to promote cell death by AMPK activation: autophagy [23,24]. Activation of AMPK directly phosphorylates and activates Ulk1 to trigger cell autophagy [23,24]. Meanwhile, AMPK-medicated mTORC1 inhibition also promotes autophagy, through removing Ulk1 inhibition by mTORC1 [23,25]. As a matter of fact, recent studies have shown that anti-cancer agents (i.e. resveratrol and ceramide) activate AMPK-dependent autophagic death pathway [26,27]. Activation of AMPK by aspirin induces autophagic cell death in colorectal cancer cells [28,29]. In the current study, we also observed a significant autophagic cell death by berberine in HepG2 cells, which was associated with mTORC1 inhibition. Activation of AMPK appeared to be important for the process, as inhibition of AMPK by RNAi or compound C suppressed autophagy induction and mTORC1 inhibition.

Activation of mTORC1 is important for HepG2 cell survival, proliferation and apoptosis resistance [30]. In the current study, we found that two mTORC1 blockers (rapamycin and RAD001) inhibited HepG2 cell survival. Interestingly, although berberine or the two inhibitors almost blocked mTORC1 activation, it simultaneously activated Akt. These results suggested that berberine-induced mTORC1 inhibition was not dependent on its effect on Akt, rather Akt activation by berberine might be due to mTORC1 or S6 inhibition [31]. Further, we provided evidence to support that mTORC1 inactivation by berberine might be associated with AMPK, as inhibition of AMPK reversed mTORC1 inhibition by berberine. It is known that AMPK inhibits mTORC1 activation through the following two mechanisms: by phosphorylation and activation of TSC2 (tuberous sclerosis protein 2), the mTOR inhibitory protein [32], or by phosphorylation of Raptor (regulatory associated protein of mTOR) [33].

It should be noted that AMPK inhibition only reduced, but not reversed HepG2 cytotoxicity-induced by berberine. This could be due to the incomplete inhibition of AMPK by the methods used in this study (RNAi or compound C). However, it is more likely that AMPK activation is among many mechanisms activated by berberine to mediate HepG2 cell death [5]. Other signals independent of AMPK activation are likely to participate in the process [5]. Meanwhile, although studies including this study have confirmed AMPK activation by berberine, the potential upstream signal for this activation is not known.

## Conclusions

Together, these results suggested that berberine-induced both apoptotic and autophagic death requires AMPK activation in HepG2 cells.

## Methods

### Chemicals and reagents

Berberine hydrochloride, 5-aminoimidazole-4-carboxyamide-1-β-D-ribofuranoside (AICAR), 3-methyladenine (3-MA), Bafilomycin A1, (Baf A1), NH_4_Cl and mouse monoclonal β-actin antibody were purchased from Sigma (Louis, MO). Z-VAD-fmk, compound C, rapamycin and RAD001 were purchased from Calbiochem (Darmstadt, Germany). Anti-Erk1/2 and Akt, AMPK, ACC and S6 antibodies were purchased from Santa Cruz Biotechnology (Santa Cruz, CA). All other phospho (p)- and non-phospho-antibodies were purchased from Cell Signaling Technology (Bevery, MA).

### Cell culture

The HepG2 cell was obtained from Chinese Academy of Sciences Cell Bank (Shanghai, China). Cells were maintained in DMEM medium (Sigma), supplemented with a 10% fetal bovine serum (FBS, Invitrogen, Carlsbad, CA), Penicillin/Streptomycin (1:100, Sigma, St. Louis, MO) and 4 mM L-glutamine (Sigma), in a CO_2_ incubator at 37°C.

### Cell viability assay

Cell viability was measured by the 3-[4,5-dimethylthylthiazol-2-yl]-2,5 diphenyltetrazolium bromide (MTT, Sigma) assay as described before [34].

### BrdU incorporation assay

HepG2 cells were seeded at a density of 1 × 10^5^ cells/well in 0.5 ml DMEM containing 10% FBS onto the 48-well tissue culture plates, cells were serum-starved for 24 hours and then exposed to various concentrations of Berberine for 48 hours. The cell proliferation was assessed using BrdU incorporation though the BrdU ELISA colorimetric assay (Roche, Indianapolis, IN) according to the manufacturer’s protocol. The ELISA OD value of treatment group was normalized to that of untreated control group. Each condition was tested in triplicate.

### Cell apoptosis assay

HepG2 cell apoptosis was detected by the Annexin V Apoptosis Detection Kit (Beyotime, Shanghai, China) according to the manufacturer’s protocol. Briefly, one million HepG2 cells with indicated treatment were stained with FITC-Annexin V and propidium iodide (PI) (Beyotime, Shanghai, China). Both early (annexin V^+^/PI^−^) and late (annexin V^+^/PI^+^) apoptotic cells were sorted by a fluorescence-activated cell sorting (FACS) machine (Becton Dickinson FACS Calibur).

### Trypan blue staining

The number of “dead” HepG2 cells (trypan blue dye positive) after indicated treatment was recorded, and the percentage of death HepG2 cells was calculated by the number of the trypan blue dye positive cells divided by the total number of the cells.

### Quantification of autophagic cells

HepG2 cells were transfected with GFP-light chain 3 (LC3) in the pcDNA3 plasmid using Lipofectamine 2000 (Invitrogen, USA) in serum- and antibiotic-free medium for 6 hours, followed by a 72 hours incubation in growth medium (with FBS). Afterwards, cells were selected with 1 mg/ml G418 (Gibco, USA) to establish a stable cell line expressing the GFP-LC3 fusion protein. Selected cells were seeded onto confocal cover-slips and treated as described in figure legends. The accumulation of GFP-LC3 was examined by fluorescence microscopy. Autophagic cells were recorded by counting the percentage of cells showing an accumulation of intense GFP-LC3 puncta, analyzing 100 cells per preparation in three independent experiments.

### Western blot assay

As described before [34], aliquots of 30–40 μg of proteins from each sample (treated as indicated in the legends) were separated by 10% SDS–polyacrylamide gel electrophoresis (SDS-PAGE), and transferred onto a polyvinylidene difluoride (PVDF) membrane (Millipore, Bedford, MA). After blocking with 10% of milk for 1 hour at room temperature, the PVDF membrane was incubated with the indicated primary antibody overnight at 4°C, followed by incubation with corresponding secondary antibody for 30 min to 1 hour at room temperature. Antibody binding was detected with the enhanced chemiluminescence (ECL) detection system (Amersham Biosciences, Piscataway, NJ). The intensity of indicated band was quantified using Image J software (http://rsbweb.nih.gov/ij/download.html), and the value was normalized to corresponding loading control, and was expressed as fold change vs. control group.

### RNA interference (RNAi)

The RNAi sequences (5′GCAUAUGCUGCAGGUAGAU3′ [35] and 5′AAGGAAAGTGAAGGTGGGCAA3′ [36]) against human AMPK-α1/2 were synthesized by GENEWIZ, Inc. (Suzhou, China). Non-sense control RNAi was purchased from Santa Cruz and was used as RNAi-negative control. Beclin-1 siRNA and LC3B siRNA were purchased from Cell Signaling Tech (Shanghai, China). Transfection was performed as described before [37]. Briefly, HepG2 cells were cultured on a six-well plate with 60% confluence in antibiotic- and serum-free medium. Targeted and control RNAi (100 μM) and 3.0 μl of Lipofectamine PLUS Reagent (Invitrogen, Carlsbad, CA) were diluted in 90 μl of siRNA dilution buffer (Santa Cruz). To this was added 3 μl of Lipofectamine LTX. The transfection complex was then added to the well containing 1 ml of DMEM for 12 hours, with a final RNAi concentration of 100 nM. Growth medium was then added back to the cells, which were cultured for additional 48 hours. Expression level of target proteins in transfected cells was always tested by western blots. Only cells with target protein significant-knockdown were used for experiments.

### Statistics analysis

All data were normalized to control values of each assay and were presented as mean ± standard deviation (SD). Data were analyzed by one-way ANOVA followed by a Scheffe’s f-test by using SPSS software (SPSS Inc., Chicago, IL, USA). Significance was chosen as ***p*** < 0.05.

## Abbreviations

3-MA: 3-methyaldenine; ACC: Acetyl-coA carboxylase; AMPK: AMP-activated protein kinase; AICAR: 5-aminoimidazole-4-carboxamide ribotide; JNK: c-Jun N-terminal kinase; LC3B: Light chain 3B; MTT: 3-[4,5-dimethylthylthiazol-2-yl]-2,5 diphenyltetrazolium bromide; mTOR: Mammalian target or rapamycin; mTORC1: mTOR complex 1; PI: Propidium iodide; RNAi: RNA interference.

## Competing interests

The authors declare that they have no competing interests.

## Authors’ contribution

RY, LC, BW, HJ and ZZ carried out the experiments. RY, LC and LS participated in the design of the study and performed the statistical analysis. RY, LC and LS conceived of the study, and participated in its design and coordination and helped to draft the manuscript. All authors read and approved the final manuscript.
